# Research on Pathogenic Hippocampal Voxel Detection in Alzheimer's Disease Using Clustering Genetic Random Forest

**DOI:** 10.3389/fpsyt.2022.861258

**Published:** 2022-04-07

**Authors:** Wenjie Liu, Luolong Cao, Haoran Luo, Ying Wang

**Affiliations:** ^1^School of Computer Information and Engineering, Changzhou Institute of Technology, Changzhou, China; ^2^College of Intelligent Systems Science and Engineering, Harbin Engineering University, Harbin, China; ^3^School of Computer Science and Engineering, Changshu Institute of Technology, Suzhou, China

**Keywords:** Alzheimer's disease, genetic evolution, clustering evolution, random forest, voxel-based features

## Abstract

Alzheimer's disease (AD) is an age-related neurological disease, which is closely associated with hippocampus, and subdividing the hippocampus into voxels can capture subtle signals that are easily missed by region of interest (ROI) methods. Therefore, studying interpretable associations between voxels can better understand the effect of voxel set on the hippocampus and AD. In this study, by analyzing the hippocampal voxel data, we propose a novel method based on clustering genetic random forest to identify the important voxels. Specifically, we divide the left and right hippocampus into voxels to constitute the initial feature set. Moreover, the random forest is constructed using the randomly selected samples and features. The genetic evolution is used to amplify the difference in decision trees and the clustering evolution is applied to generate offspring in genetic evolution. The important voxels are the features that reach the peak classification. The results demonstrate that our method has good classification and stability. Particularly, through biological analysis of the obtained voxel set, we find that they play an important role in AD by affecting the function of the hippocampus. These discoveries demonstrate the contribution of the voxel set to AD.

## Introduction

Alzheimer's disease (AD) can severely affect a variety of cognitive functions, including memory. Since the hippocampus played an important role in memory, there was interest in the possibility that hippocampal degeneration led to an age-related reduction (1). Research on the hippocampus had mainly focused on changes in hippocampal morphology and function, such as the impact of changes in volume on AD (2, 3), and which functions of the hippocampus can be caused by changes in shape (4, 5). These studies lacked the exploration of more subtle changes in the hippocampus. Based on this problem, scientists divided the hippocampus into different hippocampal subregions (6). From the perspective of subregions, they studied which specific changes in the hippocampus led to changes in hippocampal function that eventually was related to AD (7).

In recent years, research on AD using machine learning became an important field. Jiao et al. applied the graph regularization non-negative matrix factorization to factorize the vectorized dynamic functional networks matrix and evaluated the similarity between early mild cognitive impairment (EMCI) and healthy control (HC) (8, 9). The MCI participants were divided into two groups (early MCI and late MCI) according to the severity of amnestic impairment in ANDI. Among these participants, the early MCI (EMCI) group meets the following condition: 1 Standard Deviation ≤ memory test performance—standardized norms ≤ 1.5 Standard Deviation. The late MCI (LMCI) group meets the following condition: memory test performance—standardized norms ≥ 1.5 Standard Deviation. Li et al. constructed a neural network model using magnetic resonance imaging (MRI) images and used transfer learning to train the constructed model, demonstrating for the first time that non-invasive MRI is related to the development of AD (10). Fidel et al. used genetic algorithm and support vector machine (SVM) to screen 370,750 SNPs and obtained the pathways related to colorectal cancer (11). Sun et al. proposed a multi-layer deep neural network survival model and compared the survival model based on classical machine learning. The proposed model was not only superior in accuracy to existing survival models but also could screen out effective risk groups by learning the complex structure between SNPs (12). Furthermore, there were studies based on brain functional networks and variational auto-encoder for mild cognitive impairment research (13, 14).

Due to the important association between the hippocampus and AD, the study of the hippocampus combined with machine learning was an important research field. Yi et al. outlined an application of machine learning methods to brain MRI images, and introduced commonly hippocampal segmentation methods (15). Li et al. applied the feature detection method based on SVM and leave-1-out cross-validation to classify AD and HC (16). Tsao et al. used a convex fused sparse group lasso method and multivariate tensor-based morphometry method to predict the AD features (17). Liu et al. introduced a fusion method using the deep belief network method and the lattice Boltzmann method to segment the MRI image, and the correlation and consistency were compared with manual segmentation methods (18). Using the SVM, random forest, logistic regression, and K-Nearest Neighbors, Uysal et al. analyzed the MRI images to distinguish stages of AD (19). However, these studies were focused on the whole hippocampus, and studies on subtle hippocampal voxels were lacking.

To bridge this gap, we proposed a novel method based on clustering genetic random forest to identify the important hippocampal voxels. Firstly, we processed the MRI images to obtain the voxel-based images. Then, we constructed the initial feature set using the resulting images. Subsequently, we applied the random forest, genetic evolution, and clustering evolution to calculate the classification accuracy and mine the features. The experiment results demonstrate that the identified voxels were associated with AD by affecting the function of the hippocampus.

## Materials and Methods

### Imaging Data

In this study, a total of 1,515 non-Hispanic white participants had high-quality genotype data and MRI image data in ANDI database at the same time, so they were included in the study after quality control. We downloaded 1,515 participants with MRI scans from the Alzheimer's Disease Neuroimaging Initiative (ADNI) database (adni.loni.usc.edu). Table 1 shows the characteristics of the 1,515 participants.

**Table 1:** Participant characteristics.

**Subjects**	**HC**	**EMCI**	**LMCI**	**AD**	***P***
Number	442	273	504	296	-
Gender (M/F)	223/219	153/120	309/195	166/130	<0.001
Age (mean ± sd)	74.5 ± 5.6	71.3 ± 7.1	74.0 ± 7.6	75.1 ± 5.5	<0.001
Edu (mean ± sd)	16.4 ± 2.6	16.1 ± 2.6	16.0 ± 2.9	16.3 ± 2.6	<0.001

*HC, Healthy Control; EMCI, Early Mild Cognitive Impairment; LMCI, Late Mild Cognitive Impairment; AD, Alzheimer's Disease*.

Using the T1-weighted template, we aligned the MRI scans collected by voxel-based morphometry. Then, we normalized the aligned images to the Montreal Neurological Institute space. The resulting images were segmented, extracted, and smoothed with an 8-mm FWHM kernel. The hippocampus was extracted using the Automatic Anatomical Labeling atlas (20), freesurfer 6.0 (6), and FMRIB Software Library v6.0 (21). To reduce the time for subsequent analysis, we down-sampled the original images (182 × 218 × 182) in three dimensions and obtained the images of size 61 × 73 × 61.

### Construction of Clustering Genetic Random Forest

As description in Imaging Data, we obtained the coordinate information of the left and right hippocampus, including 281 voxels in left hippocampus and 302 voxels in right hippocampus, and saved them as matrices *M* and *N* (*M* for left hippocampus and *N* for right hippocampus). Let *vm*_*i*_ and *vn*_*j*_ represent the elements of *M* and *N*. Then we combined the two matrices to get the initial feature site set *V* (*V* = [*vm*_1_, *vm*_2_, …, *vm*_*i*_, *vn*_1_, *vn*_2_, …, *vn*_*j*_], *i* = 281, *j* = 302).

**Figure 1:**
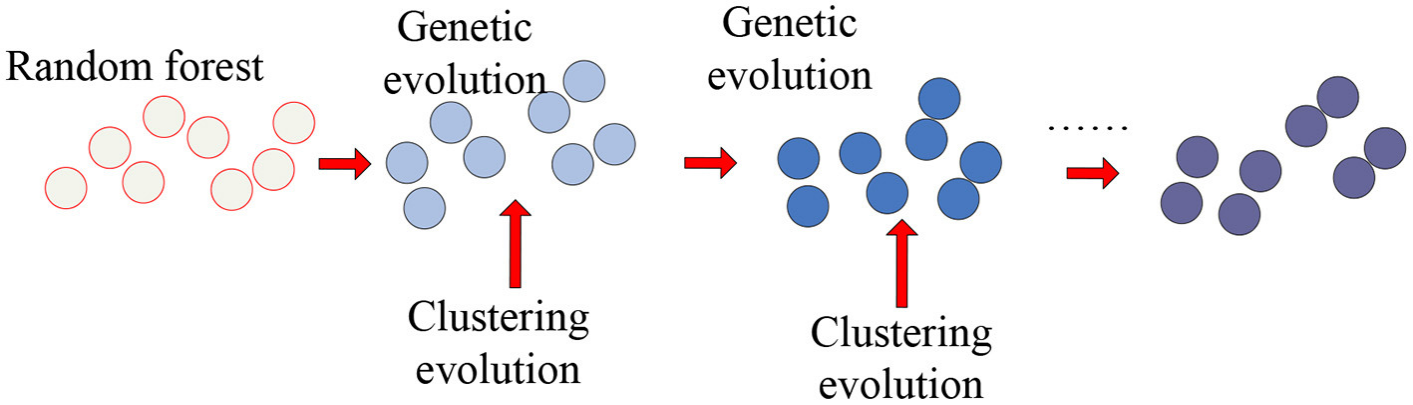
The schematic diagram of clustering genetic random forest.

As the representative classifier of machine learning, the random forest was used to identify important features from a large number of features. Therefore, we applied the clustering genetic random forest to mine the important voxel set and the random forest to obtain the initial decision trees and genetic evolution was introduced to evolve decision trees. The clustering evolution was introduced during genetic evolution to obtain new offspring. Through these steps, the features with high classification accuracy were selected from *V*. The schematic diagram is shown in Figure 1.

We used the AD and HC groups to constitute the initial dataset *S*. The *S* was defined as Equation 1.


(1) $$\begin{array}{l} S=\left\{x_{k},\;y_{k}\right\},\;k\in \left[1,\;738\right] \end{array}$$

where *x*_*k*_ is the voxels of *S*, and *y*_*k*_ is the corresponding label of *x*_*k*_ (AD is represented by −1 and HC is represented by 1).

The *S* was randomly divided into training set, validation set and test set by 6:2:2. Using the training set, we randomly selected $\sqrt{583\;}\approx \;24$ features and labels and constructed a single decision tree. Since a random forest was consisted of many decision trees, we repeated the steps above for 300 times. A random forest with 300 decision trees was formed.

The initial decision trees were regarded as the population of genetic evolution. Then, we randomly selected two groups of five trees. The Euclidean distance was introduced to calculate the similarity between trees. The Euclidean distance was defined as Equation 2.


(2) $$\begin{array}{l} D=\sqrt{\overset{n}{\underset{i=1}{\sum }}{\left(x_{1i}-x_{2i}\right)}^{2}} \end{array}$$

**Figure 2:**
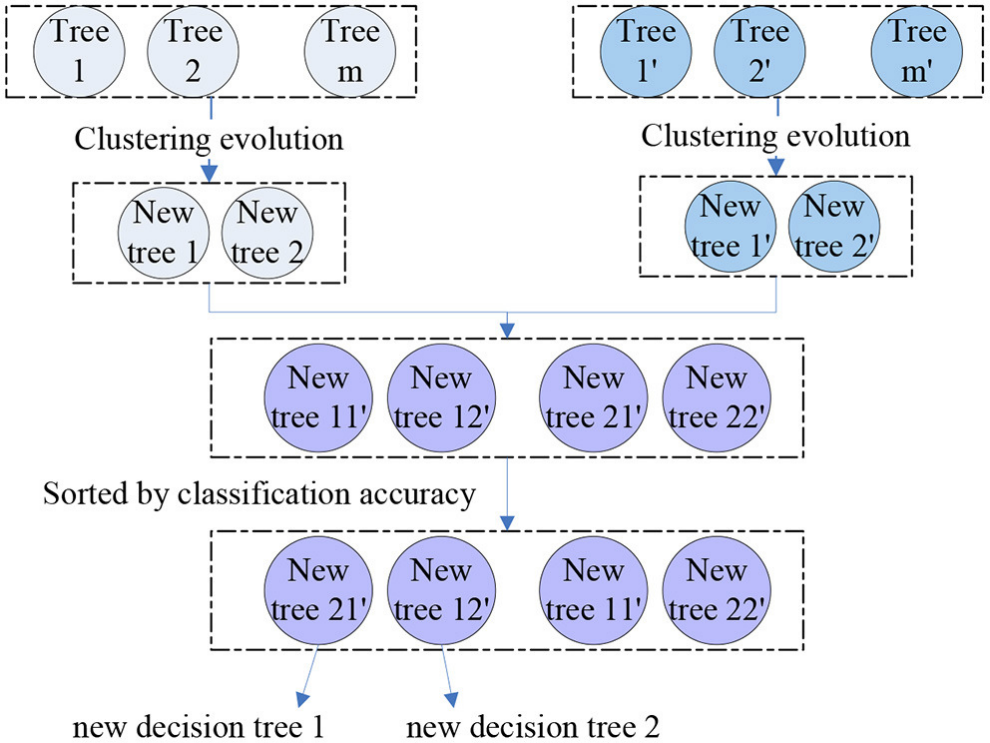
The schematic diagram of the application of clustering evolution in genetic evolution.

Subsequently, we applied the clustering evolution to identify the parents. For each group, we calculated the similarities of trees and obtained the upper triangular similarity matrix *M*_*u*_(Equation 3).


(3) $$M_{u}=\left[\begin{array}{ccc} 0 & M_{1,2} & \begin{array}{ccc} M_{1,3} & M_{1,4} & M_{1,5} \end{array}\\ 0 & 0 & \begin{array}{ccc} M_{2,3} & M_{2,4} & M_{2,5} \end{array}\\ \begin{array}{c} \begin{array}{c} 0\\ 0 \end{array}\\ 0 \end{array} & \begin{array}{c} \begin{array}{c} 0\\ 0 \end{array}\\ 0 \end{array} & \begin{array}{c} \begin{array}{ccc} \begin{array}{c} 0\\ 0 \end{array} & \;\begin{array}{c} M_{3,4}\\ 0 \end{array} & \begin{array}{c} M_{3,5}\\ M_{4,5} \end{array} \end{array}\\ \begin{array}{ccc} \;0\; & \;0\; & 0 \end{array}\; \end{array} \end{array}\right]$$

where the *M*_1, 2_ is the similarity of tree 1 and tree 2. Then, we performed clustering evolution on the decision trees and selected the tree with the best classification accuracy as parent 1. Another parent 2 was the tree with the largest distance from parent 1, i.e., the parent 1 and 2 were the resulting clusters obtained by clustering evolution. We obtained four parents from the two groups and four new offspring were generated by permutation and combination of these four parents. By calculating the classification performance of these four resulting trees, we selected the best two as the final offspring. The schematic diagram of genetic evolution and clustering evolution are shown in Figure 2. A new random forest with 300 decision trees was formed by repeating the methods above for 150 times.

Assuming that the genetic evolution reached the *n*th generation to achieve the optimal classification performance, the accuracy of the resulting decision trees at this time was defined as


(4) $$\begin{array}{l} Acc_{x}=\frac{N_{vx}}{N_{v}},\;x\in \left[1,300\right] \end{array}$$

where *Acc*_*x*_ is the final accuracy of tree *x*, *N*_*vx*_ is the number of correct predictions by tree *x* in validation set, x is the serial number of the decision trees, and *N*_*v*_ is the size of the validation set. Through the steps above, the clustering genetic random forest model was constructed.

### Parameter Optimization Adjustment

For the constructed model, the best parameter combination of the decision tree number and genetic evolution times was selected. Firstly, the decision tree number and genetic evolution times were defined in (300, 500) and (1, 500). Then, the combination of two parameters was iterated over, and the best one was the optimized parameter. To avoid the difference caused by the decision tree composed of randomly selected features, we repeated the steps above for 10 times. Finally, among the 10 results, we selected the best one as the optimized parameter.

### Important Voxel Set Determination

The accuracy of the resulting random forest was tested using the test set. The voxels sites in resulting random forest classified AD and HC, suggesting that these voxels were quite different in AD and HC. Therefore, we defined these voxels as important voxels. AD abnormal hippocampal voxels were further extracted from the important voxels. The abnormal hippocampal voxels were extracted by the following steps.

Firstly, we counted the frequency of voxels in the resulting random forest and sorted them in descending order of frequency. Then, we divided the voxels into subsets and evaluated these subsets using a traditional random forest. Subsequently, we defined the subset with peak classification accuracy as the important voxels set. Finally, we counted the frequency of voxels in the important voxels set and considered the top *N*_*f*_ voxels as the abnormal hippocampal voxels based on the frequency.

### Biological Analysis

To analyze the biological significance of the abnormal voxels, by jointly analyzing the genetic data and image data, we performed quality control (QC) and genome-wide association studies (GWAS) based on linear regression in PLINK v1.9 (22). After QC, we obtained 5,574,300 SNPs and applied the age, gender, education and the top four principal components from population stratification analysis as covariates. With the GWAS results, we applied ECS method (23) and Genome Reference Consortium Human build 37 to calculated the genes' *p*-values and obtained 10,435 genes. By applying Bonferroni correction to the resulting genes, we selected 334 genes with corrected *p*-values < 0.05 for enrichment analysis and pathway analysis (24).

## Results

### Parameter Optimization Results

**Figure 3:**
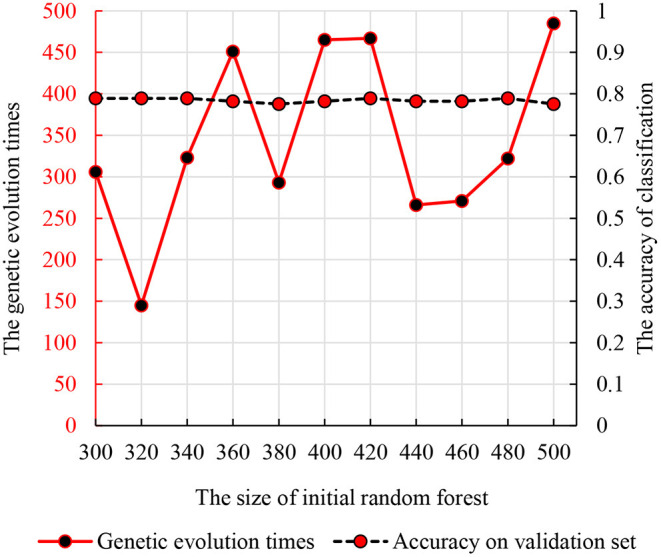
The relationship between the times of genetic evolution with clustering evolution applied and initial random forest number.

We used the strategy described in section Parameter Optimization Adjustment to identify the parameters optimization results. As mentioned previously, we set the number of decision trees in the interval of (300, 500) and the times of genetic evolution in the interval of (1, 500). Then, we compared the accuracies of all parameter combinations. Specifically, we constructed 11 random forests in the interval (300, 500). The numbers of decision trees were 300, 320, …, 500. For each initial random forest, we performed genetic clustering among 500 parameter combinations to evaluated the accuracy and identify the optimal combination. To avoid the differences caused by different features in random forests, we conducted 10 independent repeated experiments and selected the best one as the optimal combination. The accuracy and parameter combination are shown in Figure 3. From Figure 3, we find that the peak value is at 300 initial decision trees, and the corresponding genetic evolution times are 306. The optimal parameters are 300 and 306.

### Comparison With Other Methods

Besides the proposed model, we tested other three models to compare their accuracy and the three models were the traditional random forest, the clustering evolution random forest (25), and the genetic evolution random forest (26).

#### Traditional Random Forest

**Figure 4:**
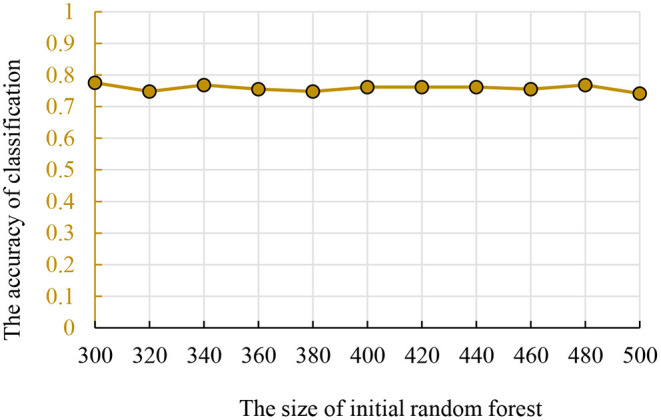
The relationship between the accuracy and initial random forest number.

The size of traditional random forest was also in the interval (300, 500). To ensure the results' comparability between different methods, we used the same dataset for training and parameter optimization. The accuracy and parameters are shown in Figure 4 and the best initial decision trees are 300.

#### Clustering Evolutionary Random Forest

**Figure 5:**
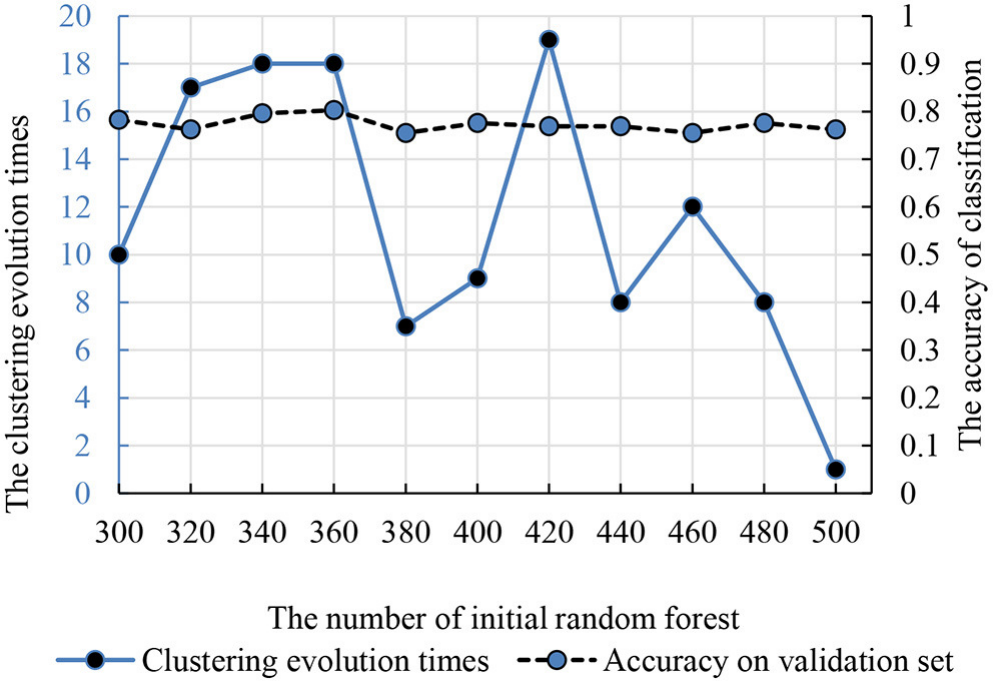
The relationship between the clustering evolution times and initial random forest number. The dotted line is the classification accuracy. The solid line is the times of clustering evolution times based on the decision trees.

Compared with the traditional random forest, the clustering evolution random forest introduced the process of clustering. Therefore, the number of initial decision trees and clustering evolution times was in the interval (300, 500) and (1, 20). The accuracy and parameters are shown in Figure 5 and the best initial decision trees and clustering evolution times are 360 and 18.

#### Genetic Evolutionary Random Forest

**Figure 6:**
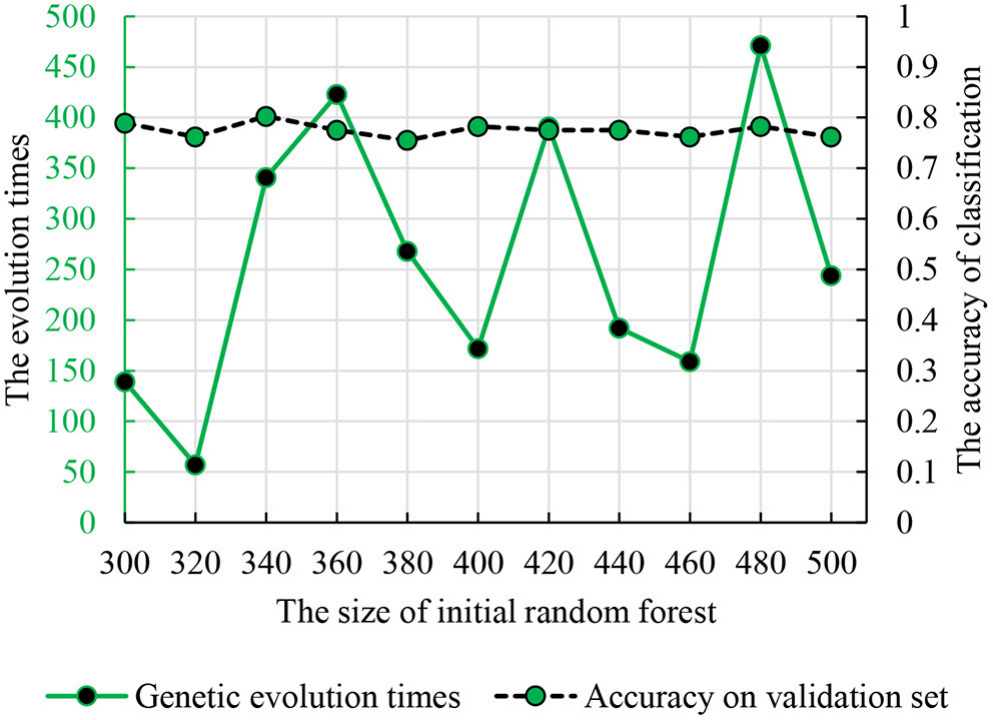
The relationship between the genetic evolution times and initial random forest number. The dotted line is the classification accuracy. The solid line is the times of genetic evolution times based on the decision trees.

To find the optimal parameters, the genetic evolution random forest introduced the genetic process and the genetic evolution times were in the interval (1, 500). Figure 6 shows the accuracy and parameter combination and the best parameter combination is 340 and 341.

#### Comparison of the Four Methods

**Figure 7:**
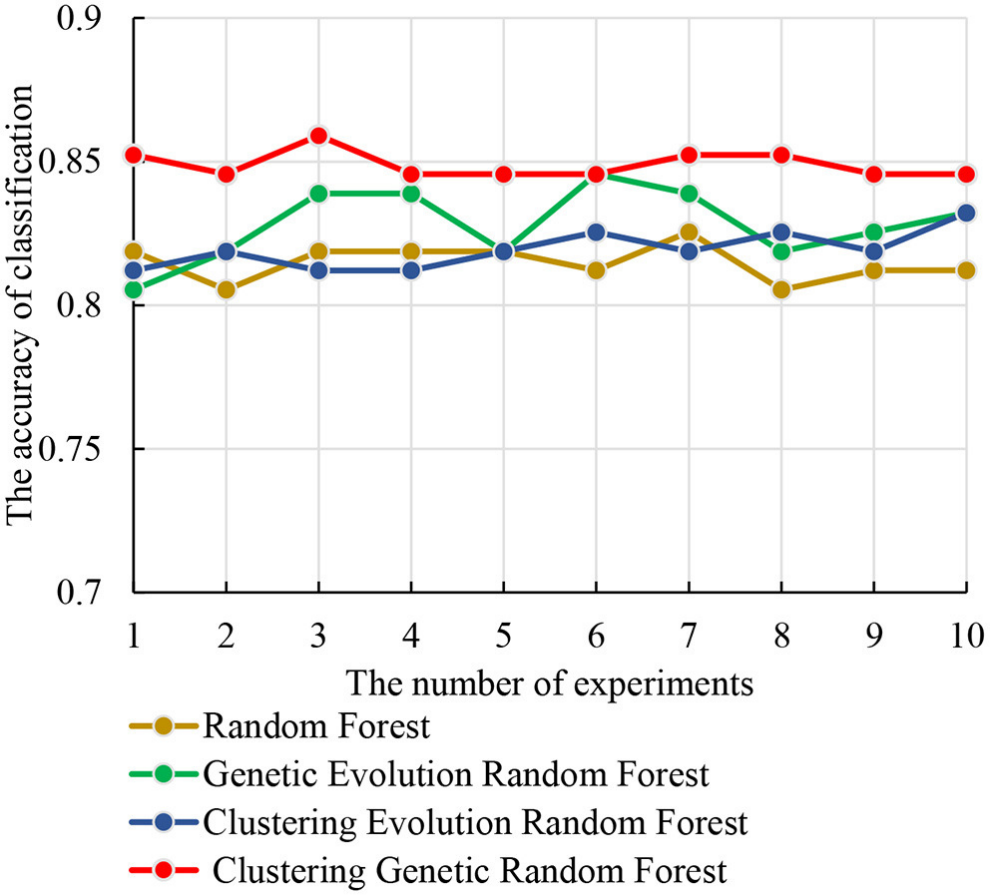
The relationship curves of accuracy and the four models in 10 independent experiments.

The test set was used to identify the classification performance of the four models. To ensure the reliability of the results, we performed 10 independent repeated experiments in each model using the optimal parameter combinations obtained above. The accuracies of the four models are shown in Figure 7. From Figure 7, we observe that the clustering genetic random forest has the best classification accuracy, and the peak is 85.91%, while other three models are all below 85%. The peak of genetic evolution random forest is 84.56% and is superior to the other two. Moreover, the stability of clustering genetic random forest is also the best among the four models and its classification accuracy differs within 1.34%. This indicates that although the features in the initial decision tree are different, the final classification accuracy difference is small after clustering genetic evolution. The results prove that the accuracy and stability are improved in our model.

### The Extraction of Abnormal Hippocampal Voxels

**Figure 8:**
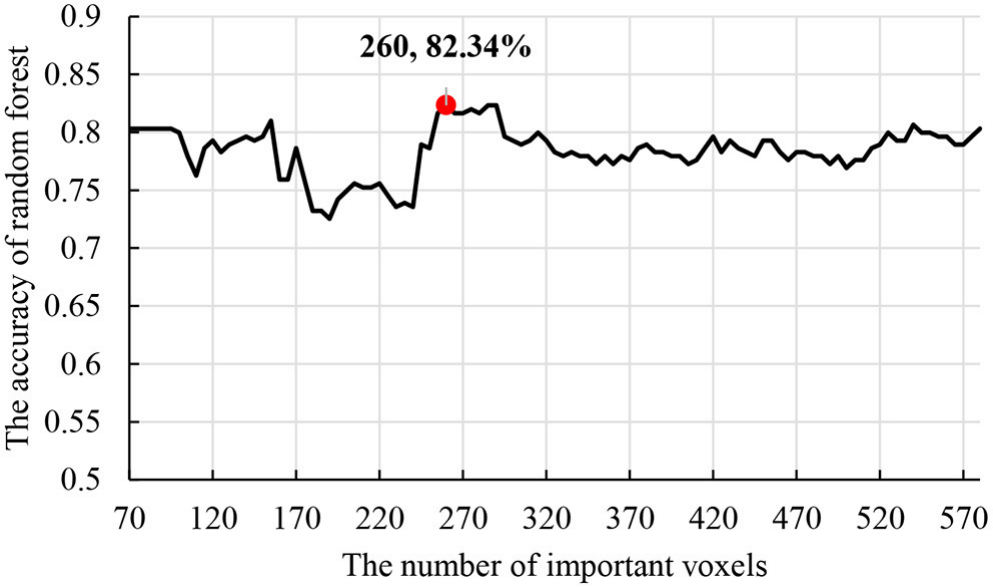
The accuracy of the random forest on different subsets.

Figure 7 shows that the clustering genetic random forest is a more effective model in classification. The essence of the identified features was hippocampal voxels. Therefore, the abnormal hippocampal voxels could be detected by analyzing the features in the resulting random forest. The voxels in resulting decision trees were candidate abnormal voxels. Table 2 lists the top 14 voxels with frequency >25. However, these voxels were not all abnormal voxels, and we needed to extract the voxels with the best classification performance from them. We firstly set the number of candidate abnormal voxels subsets to be in the interval (70, 580) with a stride of 5. The classification performance was tested using a random forest that consisted of 340 decision trees. Figure 8 shows the accuracies of the subsets and the peak accuracy is 82.34%. The subset with accuracy 82.34% was the abnormal hippocampal voxels. The top 260 voxels are in Supplementary Table 1.

**Table 2:** The important abnormal hippocampal voxels with frequency greater than 24.

**Voxel serial number**	**Frequency**	**Voxel serial number**	**Frequency**
Voxel 480	29	Voxel 135	25
Voxel 60	27	Voxel 142	25
Voxel 243	27	Voxel 147	25
Voxel 79	26	Voxel 446	25
Voxel 280	26	Voxel 494	25
Voxel 412	26	Voxel 507	25
Voxel 128	25	Voxel 557	25

We defined the abnormal hippocampal voxels according to the experiment using random forest. The subset with a high frequency was the abnormal hippocampal voxels in AD.

Table 3 shows the important abnormal hippocampal voxels discovered by four models. The fewest important voxels were identified by the genetic evolution random forest, followed by clustering genetic random forest. Interestingly, the random inheritance was applied in these two models, the obtained voxels were the least, and their ratio was the highest. Combined with Figure 7, a higher classification performance was found in the model with high ratio to our model than other two models. This indicated that the genetic process improved the classification performance.

**Table 3:** The important abnormal hippocampal voxels identified by the traditional random forest.

**Method**	**Discoveries**	**Overlap with our method**
CGRF	260	-
RF	570	252
GERF	90	44
CERF	535	238

*CGRF, clustering genetic random forest; RF, random forest; GERF, genetic evolution random forest; CERF, clustering evolution random forest*.

### Assessment of Biological Significance

**Figure 9:**
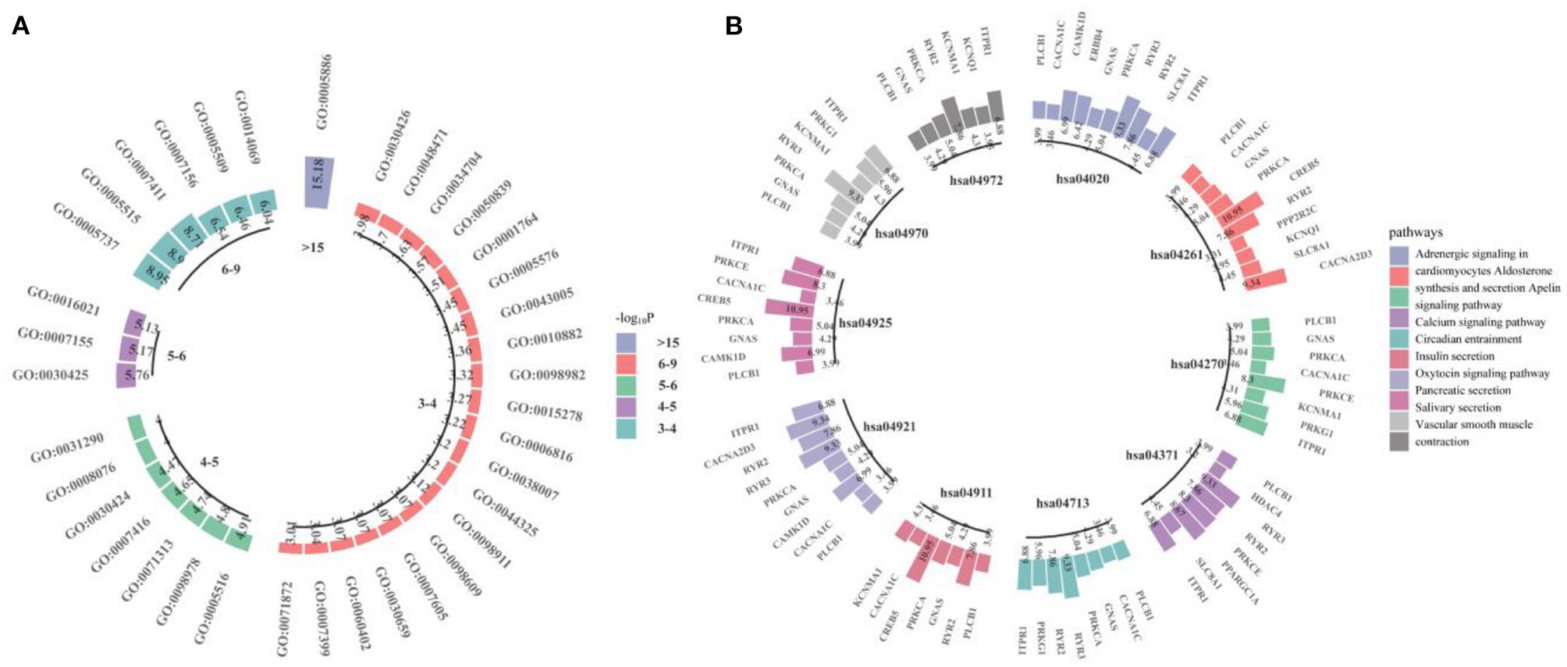
The GO terms and top 10 pathways identify by our model. **(A)** is for GO terms and **(B)** is for pathways.

We performed GWAS using the obtained 260 important voxels and gene-based association analysis using the resulting *p*-value of SNPs to identify the pathogenic genes of AD. One hundred and Fifty one genes passed the Bonferroni correction (corrected *p*-value < 0.001) and were considered as the pathogenic genes. The top 10 genes are listed in Table 4. We applied the selected genes to detect the gene ontology (GO) terms and pathways that provided information on AD pathological relationships. We identified 37 GO terms and 72 pathways and showed them in Figure 9 (https://hiplot.com.cn/basic/circular-barplot).

**Table 4:** The top 10 significant genes identified in our study.

**No**.	**CHR**	**Gene**	**Corrected *P***
1	8	*CSMD1*	4.28E-43
2	16	*RBFOX1*	1.75E-21
3	9	*PTPRD*	9.38E-20
4	16	*WWOX*	1.09E-17
5	3	*FHIT*	2.12E-16
6	11	*DLG2*	2.76E-13
7	7	*DPP6*	6.11E-13
8	11	*NELL1*	8.53E-13
9	7	*MAGI2*	1.56E-12
10	4	*SORCS2*	3.36E-12

*CHR is the chromosome; Gene is the gene name; corrected P is the p-value after Bonferroni correction*.

## Discussion

In this study, we proposed a model based on machine learning to identify the abnormal hippocampal voxels. Previous research on machine learning was used to detect features classified AD and HC (27–29). Bron et al. applied the SVM and convolutional neural network to predict the MCI patient's conversion to AD (30). Huang et al. proposed Epigenome-Wide Association Studies plus using a supervised machine learning strategy to predict the significant brain CpGs associated with AD (31). However, our model started with voxel images and discovered a voxel set distinguished AD and HC well. An interesting finding was that we introduced the clustering evolution to select the parents with low similarity. The advantages were that not only the good decision trees were preserved, but also the diversity of decision trees was guaranteed. As shown in Figure 7, we come to a conclusion that the genetic evolution was quite suitable for detecting the voxel. The combination of genetic evolution and clustering evolution could improve the classification performance and stability of the model. As shown in Table 3, the voxels identified by random forest model and clustering evolution random forest model are above 500. The voxels of our model are 260, and the voxels' overlaps with the two models are 252 and 238. They are very close to the resulting voxels of our model. This indicates that our model extracts the important voxels that contribute to classification and discards poorly classified voxels.

For the identified genes, in the *CSMD* gene family associated with AD (32, 33), *CSMD1* was expressed in developing nerve cells (34) and the expression of *CSMD1* (Corrected *P*: 4.28E-43) was associated with cognitive function (35) and mental illness (36). Its homologous gene *CSMD2* could lead to the decline of cognitive ability (37), and *CSMD3* existed in the hippocampus and was related to AD by affecting the transmission of information between cells (38). *RBFOX1* (Corrected *P*: 1.75E-21) associated with AD by affecting amyloid levels (39). The lack of *PTPRD* (Corrected *P*: 9.38E-20) led to cognitive impairment and intellectual disability (40), and *PTPRD* was associated with LMCI (41). *WWOX* (Corrected *P*: 1.09E-17) deficiency led to problems with neurodevelopment (42) and aggregation of amyloid β (43).

Detection of GO terms and pathways can provide important information of the function of the pathogenic voxels. The GO terms GO:0021675 and GO:0007417 (central nervous system development, Corrected *P*: 1.90E-02) were the sub-terms of neural system development (GO:0007399, Corrected *P*: 9.09E-04), and the GO:0007399 was associated with the neurological disorders. For example, through gene-level analysis, neural function genes were enriched in GO:0007399 (44) and in neurological disease, the significant genes were also enriched in GO:0007399 (45–47). The PI3K-Akt signaling pathway (hsa04151, Corrected *P*: 1.70E-03) and AGE-RAGE signaling pathway in diabetic complications (hsa04933, Corrected *P*: 2.30E-03) involved in the process of cell apoptosis (48, 49). Together with Calcium signaling pathway (hsa04020, Corrected *P*: 2.41E-07), they were associated with Alzheimer's disease pathway (hsa05010, Corrected *P*: 1.13E-02) and contributed to AD by disrupting intracellular calcium ions (50–52). Oxytocin signaling pathway (hsa04921, Corrected *P*: 3.19E-07) played a role in AD by protecting the nerves (53). Vascular smooth muscle contraction (hsa04270, Corrected *P*: 1.26E-06) was related with AD by affecting the neurodegeneration (54).

In this study, we proposed a novel model to mine the abnormal hippocampal voxels. This model used the decision trees as the initial feature set, and applied the genetic evolution to evolve the features. In the process of genetic evolution, the clustering evolution was introduced to identify the parents. Finally, we extracted the important voxels set from the initial features. Additionally, the results demonstrated that our model was superior to other models in terms of the accuracy and stability. The voxels set identified could be regarded as the abnormal hippocampal region. Naturally, our study had several limitations. We have conducted the research of voxel detection here. However, due to limited conditions, better results may be obtained if the mouse experiments are used to verify the results. Since genetic data and imaging data are equally important; we will continue to look for other data such as SNP, protein, and RNA to construct the fusion features for detection.

## Data Availability Statement

Publicly available datasets were analyzed in this study. This data can be found at: http://adni.loni.usc.edu/.

## Ethics Statement

Ethical approval was not provided for this study on human participants because the ethical review was applied by ADNI. We applied and obtained the access from ADNI. The patients/participants provided their written informed consent to participate in this study. Written informed consent was obtained from the individual(s) for the publication of any potentially identifiable images or data included in this article.

## Author Contributions

WL and YW led, supervised, and designed the research and wrote the article. WL, LC, HL, and YW performed features extraction and selection. WL, LC, and HL performed data pre-processing and quality control. WL did biological significance analysis. All authors reviewed, commented on, edited, and approved the manuscript.

## Funding

This research was funded by MOE (Ministry of Education in China) Project of Humanities and Social Sciences (21YJAZH091) and by National Statistical Science Research Project (2020LY074). Data collection and sharing for this project was funded by the Alzheimer's Disease Neuroimaging Initiative (ADNI) (National Institutes of Health Grant U01 AG024904) and DOD ADNI (Department of Defense award number W81XWH-12-2-0012). ADNI was funded by the National Institute on Aging, the National Institute of Biomedical Imaging and Bioengineering, and through generous contributions from the following: AbbVie, Alzheimer's Association; Alzheimer's Drug Discovery Foundation; Araclon Biotech; BioClinica, Inc.; Biogen; Bristol-Myers Squibb Company; CereSpir, Inc.; Cogstate; Eisai Inc.; Elan Pharmaceuticals, Inc.; Eli Lilly and Company; EuroImmun; F. Hoffmann-La Roche Ltd and its affiliated company Genentech, Inc.; Fujirebio; GE Healthcare; IXICO Ltd.; Janssen Alzheimer Immunotherapy Research & Development, LLC.; Johnson & Johnson Pharmaceutical Research & Development LLC.; Lumosity; Lundbeck; Merck & Co., Inc.; Meso Scale Diagnostics, LLC.; NeuroRx Research; Neurotrack Technologies; Novartis Pharmaceuticals Corporation; Pfizer Inc.; Piramal Imaging; Servier; Takeda Pharmaceutical Company; and Transition Therapeutics. The Canadian Institutes of Health Research is providing funds to support ADNI clinical sites in Canada. Private sector contributions are facilitated by the Foundation for the National Institutes of Health (www.fnih.org). The grantee organization is the Northern California Institute for Research and Education, and the study is coordinated by the Alzheimer's Therapeutic Research Institute at the University of Southern California. ADNI data are disseminated by the Laboratory for Neuro Imaging at the University of Southern California. The commercial funders were not involved in the study design, collection, analysis, interpretation of data, the writing of this article or the decision to submit it for publication.

## Conflict of Interest

The authors declare that the research was conducted in the absence of any commercial or financial relationships that could be construed as a potential conflict of interest.

## Publisher's Note

All claims expressed in this article are solely those of the authors and do not necessarily represent those of their affiliated organizations, or those of the publisher, the editors and the reviewers. Any product that may be evaluated in this article, or claim that may be made by its manufacturer, is not guaranteed or endorsed by the publisher.
